# Resilience conferred by APOE-R136S: a defense bestowed by nature to combat Alzheimer’s disease

**DOI:** 10.1038/s41392-024-01775-7

**Published:** 2024-02-29

**Authors:** Bin Xiao, Joshua Kuruvilla, Eng-King Tan

**Affiliations:** 1 Department of Neurology, National Neuroscience Institute, Singapore, Singapore; 2 Neuroscience Academic Clinical Program, Duke-NUS Medical School, Singapore, Singapore; 3 Neuroscience and Behavioural Disorders Program, Duke-NUS Medical School, Singapore, Singapore

**Keywords:** Diseases of the nervous system, Molecular medicine

In two recent articles published in *Nature Neuroscience* and *Cell*, Nelson et al.^1^ and Chen et al.^2^ have shown that a rare APOE3 Christchurch (APOE3Ch) variant, R136S, has protective effects on neurodegeneration in various Alzheimer’s disease (AD) model systems through attenuating tau pathology and regulating its related neuroimmune responses. Nelson et al. and Chen et al. have provided evidence that identifying the pathophysiologic clues and potential molecular targets from a protective variant such as APOE R136S, can be an attractive viable approach to develop AD therapeutics.

Targeting amyloid-β (Aβ) and tau, the primary components of the extracellular plaques and intracellular neurofibrillary tangles that accumulate in the diseased brain has been a commonly used strategic approach to tackle AD. Although recent clinical trials of Aβ-targeting immunotherapies demonstrated significant clearance of Aβ load, the clinical outcome in patients has been variable and more data are still needed.

The two studies were initiated by the clinical observation of an APOE3Ch carrier having remarkable resistance to amyloid-associated tau pathology and cognitive decline in a known familial AD (FAD) PSEN1 E280A Colombian kindred.^3^ In the first study, Nelson et al.^1^ investigated the neuroprotection of the R136S mutation in the context of the common APOE4-related late-onset AD (LOAD), though the clinical benefits in the resilient APOE3Ch carrier were found to be against early-onset AD (EOAD) caused by PSEN1-E280A mutation. First, the authors generated human APOE4-R136S-knock-in mice before crossing them with PS19 tauopathy mice. The homozygous R136S mutation attenuated APOE4-driven accumulation of phosphorylated tau in the mice at 10 months of age. Consistently, in APOE4 isogenic human induced pluripotent stem cell (hiPSC) models with the R136S mutation generated with CRISPR–Cas-9-mediated gene editing, the authors observed similar effect of R136S mutation on the accumulation of phosphorylated tau in human neurons. On the mechanistic level, APOE4-R136S has a defective heparan sulfate proteoglycan (HSPG) binding, leading to reduced neuronal tau uptake. In addition, the mutation reduced microgliosis in tauopathy mice, and single-nucleus RNA sequencing (snRNA-seq) in these transgenic mice revealed that the R136S mutation is associated with increased disease-protective subpopulations of neurons, astrocytes and microglia and decreased subsets of oligodendrocytes, astrocytes, and microglia linked to disease, suggesting that it may play a role in mitigating APOE4-related tauopathy.

In the second study, Chen et al.^2^ characterized the APOE3Ch variant in the transgenic APP/PS1:APOE3Ch mice, by crossing APOE3Ch knock-in mice with an Aβ-depositing mice which expressed mutated human amyloid precursor protein (APP) and presenilin 1 (PS1). AD-tau brain extract was injected into these mice, and it was found that the APOE3Ch variant rescued Aβ pathology and markedly reduced tau seeding and spreading, particularly in the regions of peri-amyloid plaques. Mechanistically, reduced APOE3Ch binding to HSPG was also found in the study, triggering increased myeloid cell phagocytosis and degradation of tau aggregates. In addition, microglial response increased around the amyloid plaques, with upregulation of microglial CD68+, indicating that Aβ-mediated activation of disease-protective microglia with enhanced phago-lysosome reactivity is responsible for the reduced Aβ-induced tau pathologies. In contrast, disease-associated microglia (DAM)-associated marker, CLEC7A, around Aβ plaques was not altered, suggesting that APOE3Ch-related immune responses specifically suppress Aβ-related pathology while inducing minimum detrimental effects that may exacerbate neurodegeneration.

**Fig. 1:**
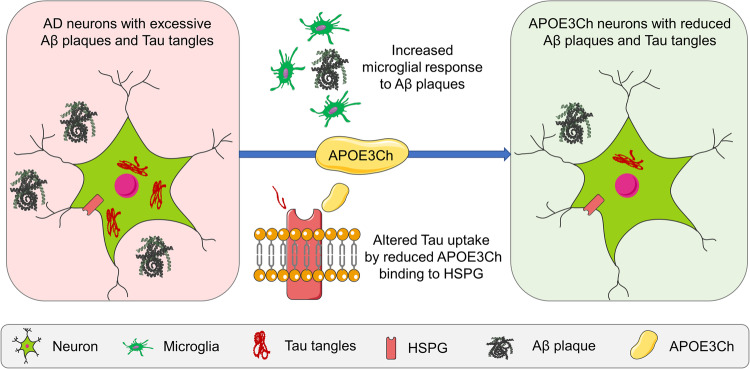
APOE3Ch attenuates AD pathology by modulating its HSPG binding and neuroimmune responses. In transgenic mouse models and hiPSC models, APOE3Ch rescued amyloid load and Tau pathology, probably by modulating HSPG-mediated Tau uptake and disease-related microglial activation. Figure was partially generated using templates from Servier Medical Art (https://smart.servier.com), which is licensed under a Creative Commons Attribution 3.0 Unported License

The two studies presented corroborative evidence that the R136S mutation indeed affords defense against AD-related neurodegeneration. The mechanistic studies in the two papers also shed light on the underpinning factors that mediated this neuroprotection, including modulated HSPG-mediated tau uptake and degradation, and microglial immune response (Fig. 1).

Future studies utilizing spatial transcriptomics may be able to better profile the gene expression adjacent to the amyloid plaques and tau neurofibrillary tangles in an unbiased manner while preserving their spatial information, and this approach has advantages over the snRNA-seq and selective immunofluorescence staining used in the two papers. Additionally, further clinical characterization of heterozygous APOE3Ch carriers is warranted, considering heterozygous R136S mutation provided partial protection against APOE4-driven neurodegeneration and neuroinflammation shown by Nelson et al.^1^ The push towards identification and utilization of natural resilience towards AD could be complemented further by studying other protective genetic variants for therapeutic purposes, including a RELN-COLBOS variant which upregulates RELN signaling to induce neuroprotection by activating its canonical protein target Dab1.^4^ Of note, the RELN-COLBOS variant influences the binding of RELN to glycosaminoglycan (GAG), the primary component of HSPG, potentially sharing a common pathway with the APOE3Ch variant. The findings collectively underscore the role of HSPG in the molecular mechanisms underpinning AD, thereby positing HSPG as a prospective therapeutic target for AD. Interestingly, a 50% risk reduction of AD development has been associated with the APOE3-Jacksonville (V236E) variant and APOE4 R251G variant,^5^ and the protective mechanisms arising from a single amino acid substitution in R251G variant should be explored. It is also interesting to investigate if additional benefits could be obtained by combining the protective *APOE* gene variants. Chen et al. found that amyloid load was reduced in the APP/PS1:APOE3Ch mice. However, Aβ burden has been shown to be high in the resilient individual with PSEN1-E280A mutation, suggesting that the protective effects of APOE3Ch is independent of Aβ, or independent of PSEN1-E280A mutation-related Aβ. The discrepancy in amyloid findings between the experimental model and the clinical case may be attributed to the species- and tissue-specific effects. While it is crucial to confirm the effects of APOE3Ch on amyloid load, it is conceivable that the APOE3Ch variant acts via other alternative pathways to mitigate neurodegeneration.

In summary, these two recent studies elucidated specific cellular and molecular pathways affected by the APOE3Ch variant, shedding light on potential targets that may confer neuroprotection in a larger group of AD patients. Specifically, cutting-edge translational AD models were employed to demonstrate the neuroprotection in the context of Aβ load, tau pathology and immune response. Should the findings of the studies be successfully replicated and further elaborated upon in pre-clinical and clinical studies, identifying druggable targets of the APOE3Ch variant could well represent an exciting novel therapeutic approach in AD.
